# Beneficial Influence of Soybean Lecithin Nanoparticles on Rooster Frozen–Thawed Semen Quality and Fertility

**DOI:** 10.3390/ani11061769

**Published:** 2021-06-13

**Authors:** Lingwei Sun, Mengqian He, Caifeng Wu, Shushan Zhang, Jianjun Dai, Defu Zhang

**Affiliations:** 1 Institute of Animal Science and Veterinary Medicine, Shanghai Academy of Agricultural Sciences, Shanghai 201106, China; sunlingwei1987@126.com (L.S.); he1037247863@163.com (M.H.); wucaifengwcf@163.com (C.W.); smalltreexj@126.com (S.Z.); 2 Division of Animal Genetic Engineering, Shanghai Municipal Key Laboratory of Agri-Genetics and Breeding, Shanghai 201106, China

**Keywords:** rooster semen, soybean lecithin, cryopreservation, artificial insemination

## Abstract

**Simple Summary:**

Soy lecithin (SL) can be used in to prevent spermatozoa cryodamage during cryopreservation by mitigating the efflux of cholesterol or phospholipids, thus reducing the formation of intracellular ice crystals. SL nanoparticles (nano-SL) have a smaller particle size and higher solubilizing capacity as compared with those that have not undergone nanotreatment. Thus, they allow for a better interaction or coating of sperm, decreasing cold shock injury during freezing–thawing processes. The objective of this study was to determine the optimal concentration of nano-SL. In order to achieve this, we assessed the quality of frozen–thawed semen in vitro and in vivo. We found that a nano-SL dosage of 1.0% in the semen extender had an affirmative influence on post-thawing quality in roosters, improving various parameters related to sperm motion, protecting the membrane and acrosome integrities, increasing mitochondrial activity and antioxidant capacity, and reducing the oxidative stress caused by the cryopreservation process. Moreover, enrichment of 1.0% nano-SL in the semen extender improved the fertilizing capacity of rooster sperm after artificial insemination.

**Abstract:**

The present study aimed to investigate the impact of different concentrations (0%, 0.5%, 1.0%, 1.5%, and 2.0%) of nano-soybean lecithin (SL) in the extender on sperm quality, sperm motion characteristics, and fertility outcomes of post-thawed rooster semen. Adult Ross broiler breeder roosters (n = 20) were subjected to semen collections twice a week for three weeks. At each collection, semen samples were pooled and allocated into five treatments corresponding to different nano-SL concentrations (control, SL0.5, SL1.0, SL1.5, and SL2.0). Sperm parameters, including motility (collected using a computer-assisted sperm analysis system), plasma membrane and acrosome integrities, and mitochondrial activity were assessed. Sperm malondialdehyde (MDA) and antioxidant activities (total antioxidant capacity (TAC); superoxide dismutase (SOD); glutathione peroxidase (GPx)) were evaluated. The fertility and hatchability obtained with frozen–thawed rooster semen supplemented with the optimum nano-SL concentration were assessed after artificial insemination. The results showed that the addition of 1% nano-SL into the extender led to a higher semen motility in roosters, improved plasma membrane and acrosome integrities, and higher mitochondrial activity of post-thawed rooster semen in comparison to controls (*p* < 0.05). The MDA levels in the SL0.5 and SL1.0 groups were lower than the other groups (*p* < 0.05). TAC activities in SL0.5, SL1.0, and SL1.5 groups were significantly higher than those in the other groups (*p* < 0.05). It was observed that the concentration of SOD was higher in the SL1.0 group than in the other groups (*p* < 0.05). The activity of GPx was not influenced in any of the cases (*p* > 0.05). Moreover, the percentages of fertility and hatchability in the SL1.0 group were higher (56.36% and 58.06%) than those in the control group (42.72% and 40.43%). In summary, the addition of nano-SL to the extenders enhanced the post-thawed semen quality and fertility of roosters by reducing the level of oxidative stress. The optimum nano-SL concentration was 1.0%. These results may be beneficial for improving the efficacy of semen cryopreservation procedures in poultry breeding.

## 1. Introduction

Sperm freezing is crucial for the preservation of biodiversity in poultry. In poultry in particular, the use of frozen semen may decrease the risk of breed and population extinctions and reduce the cost of transportation [1]. The cryopreservation of bird semen can also reduce the costs associated with feeding a large number of roosters, and increase the diffusion and measurement of genetic progress [2]. However, cryopreservation has detrimental effects on sperm viability and motility, and consequently decreases fertility. 

Artificial insemination (AI) with frozen–thawed sperm is associated with low fertility rates, which is also a central problem in commercial applications and the conservation of genetic resources in poultry [3]. Bird spermatozoa are thin and long cells and have a relatively long tail (80–90 µm) as compared to bull spermatozoa (50–60 µm) [4]. Therefore, the morphology of poultry sperm makes them more susceptible to injury during pipetting and centrifugation for semen cryopreservation. Furthermore, the head of avian spermatozoa has a lower cytoplasmic volume, which implies that it is less capable of moving in cryoprotectant agents [5]. As an indication of sperm freezability, the fluidity of poultry sperm was verified to be positively correlated with the freezing tolerance of spermatozoa. Moreover, poultry sperm membranes are enriched in polyunsaturated fatty acids, as compared to mammalian sperm. Hence, poultry sperm is more susceptible to cold stress in cryopreservation [6]. At present, many studies are being carried out to develop different freezing protocols for cryopreserving bird semen [1]. However, semen cryopreservation is in its experimental phase, and large-scale commercial applications and the conservation of genetic resources in the poultry industry remain far away. 

Soy lecithin (SL), which is mainly composed of phosphatidylcholine, has the capacity to protect the integrity of the phospholipid membrane during cryopreservation [7]. It also acts as an antioxidant against free radical damage, resulting in improved viability and motility after cryopreservation. Many studies confirm the beneficial impact of SL for semen cryopreservation in bulls [8], rams [7], canines [9], bears [10], and so on. A previous study from our laboratory demonstrated that 20% egg yolk and 2.0% SL added to the extenders produced similar protective effects on goat semen cryopreservation [11]. However, the use of SL in the extender was also reported to have a negative impact on sperm motility [12] and mitochondrial activity [13] in ram semen. 

The particle size of SL in the extenders is generally considered to be an important factor in frozen–thawed semen quality [14]. SL nanoparticles (nano-SL) have a smaller particle size and higher solubilizing capacity as compared to those that have not undergone nanotreatment; thus, they allow for a better interaction or coating of sperm, decreasing cold shock injury during freezing–thawing processes [7]. Furthermore, it was revealed that an increase in the antioxidant capacity of semen after freezing and thawing is associated with a decrease in the SL particle size [14].

As far as we are aware, the physiological mechanism of the nano-SL-based extender in the cryopreservation of rooster semen has yet to be systematically explored. As such, this study aimed to explore the influence of nano-SL at various concentrations on sperm motion parameters, plasma membrane integrity, acrosome integrity, and mitochondria function of post-thawed rooster sperm. Considering the antioxidative effect of SL in the extender, we evaluated changes in the enzymatic antioxidant activity of post-thawed sperm, including the malondialdehyde (MDA) level, the total antioxidant capacity (TAC), and the superoxide dismutase (SOD) and glutathione peroxidase (GPx) levels. Importantly, the fertility and hatchability rates after AI with frozen–thawed semen were also assessed.

## 2. Materials and Methods

In all animal experiments, the animal procedures were approved by the Ethics Committee on the Use of Animals of Shanghai Academy of Agricultural Sciences, China (Approval ID: SAASPZ0520017). All materials were acquired from Sigma Chemical Co. (St Louis, MO, USA) unless mentioned otherwise.

### 2.1. Semen Collection

Semen was collected from Ross broiler breeder roosters (n = 20; 28 weeks old), which were bred at the Shanghai Academy of Agricultural Sciences facilities. All roosters were kept in separate cages (size = 70 cm × 70 cm × 85 cm), maintained in a 15 h light:19 h dark photoperiod, fed basal diets, and had free access to water.

Semen was collected twice a week for a 3-week period (six replicates), using the dorsoabdominal massage method [15]. From the 110 ejaculates collected in this study, 95 ejaculates were used for testing, and 15 ejaculates did not meet the inclusion criteria described below and were excluded from the samples. Semen volume was measured by weighing the tubes. Ejaculates that met the standard criteria (>0.2 mL volume, ≥4 × 10^9^ spermatozoa/mL concentration, ≥ 80% sperm motility, ≥90% sperm viability, and ≤ 10% abnormal morphology) were accepted for further processing. The semen samples were pooled to eliminate individual differences and then grouped in five treatments according to different SL concentrations (0%, 0.5%, 1.0%, 1.5%, and 2.0%). The nano-SL concentration chosen for this study was chosen based on a former study [16,17].

### 2.2. Nanoparticles

The SL nanoparticles was prepared using the reverse-phase evaporation method, according to the literature, with minor modifications [14]. In total, 50 mg of SL were weighed and dissolved in 8 mL of ethanol in a round-bottom flask. The solvent was dried at 50 °C using a rotary vacuum concentrator. Then, the dried thin film was hydrated with the addition of 10 mL phosphate-buffered solution (pH = 7.4) and vortexed for 1 h to obtain a milky aqueous suspension.

The coarse emulsion was homogenized by sonication (JY92-2D, Xinyi Sonication Equipment Company, Ningbo, China; 130 W, 20 kHz,) for 15 min to reduce the size of the droplets, and then transferred into a high pressure homogenizer (Donghua High Pressure Homogenizer Factory, Shanghai, China) at 30,000 lb/in2 for 30 cycles at 4 °C. The particle sizes of the prepared nanoparticles were measured by transmission electron microscopy. SL nanoparticles with a certified particle size distribution of <100 nm were used and stored at 4 °C for future use in the semen extender.

### 2.3. Semen Extension and Freezing

Semen aliquots were diluted to a definitive concentration of 1.5 × 10^9^ spermatozoa/mL using modified Lake pre-freezing extender [18], supplemented with different levels of nano-SL (control, SL0.5, SL1.0, SL1.5, SL2.0, SL2.5). Briefly, the Lake’s extender was composed of 1000 mL sterile distilled water, 8 g D-fructose (44.4 mM), 5 g potassium acetate (50.9 mM), 19.2 g sodium glutamate (113.5 mM), 3 g polyvinylpyrrolidone (8.3 µM), 0.7 g magnesium acetate (4.9 mM), and 3.75 g glycine (50 mM). The pH was 7.0 and the osmolality was 340 mOsm/kg. The diluted semen samples were incubated 5 °C for 1 h. Then, semen samples were further diluted to 1 × 10^9^ sperm/mL with Lake pre-freezing extender containing 18% dimethylacetamide (DMA), leaving to 6% final DMA concentration. After equilibration (15 min, 5 °C), the extended semen was aspirated into 0.25 mL plastic straws, and frozen 4 cm over liquid nitrogen for 20 min. Finally, the straws were plunged and kept in liquid nitrogen for at least 1 month. For the post-thaw sperm quality assessments, frozen samples were thawed separately in a 37 °C sterile water bath for 20 s.

### 2.4. Fresh and Post-Thawed Semen Evaluation

#### 2.4.1. Sperm Concentration and Abnormal Morphology

Sperm concentration was assessed using a haemocytometer (Bürker-Türk) and expressed as 10^6^ spermatozoa/mL. Sperm morphology was assessed using Hancock solution (426 mM sodium, 21.4 mM formalin, 304.29 mM Na_2_HPO_4,_ and 99.42 mM K_2_HPO_4_) [19]. For this analysis, 10 µL of semen sample was added to 1 mL of this solution. For each sample, at least 200 spermatozoa were assessed and the percentage of morphologically abnormal sperm was analyzed using phase-contrast microscopy.

#### 2.4.2. Motility Parameters

The sperm motion was evaluated with a computer-assisted sperm assessment (CASA, IVOS Sperm Analyzer, Hamilton Thorn, Danvers, MA, USA) as described previously [20]. Samples were first diluted at 1:7 with PBS prior to measurements. A total of 5 µL of diluted semen were added onto a prewarmed (37 °C) chamber slide. For each sample, a minimum of five fields and at least 300 spermatozoa were counted. The following variables were analyzed: the total motility (MOT), progressive motility (PMOT), curvilinear velocity (VCL), straight line velocity (VSL), average path velocity (VAP), linearity (LIN), straightness (STR), amplitude of the lateral head displacement (ALH), and beat cross frequency (BCF).

#### 2.4.3. Plasma Membrane Functionality

Plasma membrane integrity was evaluated using the hypo-osmotic swelling test (HOS-t) [21]. In brief, 10 µL of semen samples were incubated in 100 µL of HOS solution at 37 °C for 60 min. The spermatozoa were fixed with formaldehyde (18.5%, 0.1 mL) after incubation. At least 200 spermatozoa from each sample were evaluated under a phase-contrast microscope at 40× magnification. The sperm that had swollen or coiled tails were considered to be HOS+. 

#### 2.4.4. Acrosome Integrity

Acrosomal status was evaluated by staining with propidium iodide (PI) and fluorescein isothiocyanate-labeled peanut agglutinin (FITC-PNA), according to the method described by Thananurak et al. [22]. The staining solution consisted of 100 µL of sodium citrate (3% diluted in NaCl 0.9%), 1 µL of PI (0.5 mg/mL), and 1.5 µL of FITC-PNA solution (1 mg/mL in PBS). For each sample, an aliquot of 500 µL of sperm suspension (1 × 10^6^ spermatozoa/mL) was centrifuged for 3 min at 1200× *g*. A total of 5 µL of sperm pellet were added to 100 µL ethanol (purity: 96%). Next, a 10 µL sample droplet was placed on a glass slide and heated in order to evaporate the solvent. Afterwards, 30 µL of work solution were added and incubated in the dark at room temperature for 5 min. After incubation, the slides were washed and examined with a fluorescence microscope (magnification, ×400). At least 200 spermatozoa per slide were observed. Sperms with PI were considered to be dead and sperms without PI were considered to be alive. Alive cells were categorized as acrosome-reacted (FITC-PNA-) or as acrosome-intact (FITC-PNA+). 

#### 2.4.5. Mitochondrial Activity

Mitochondria membrane potential was assayed using the cationic agent Rhodamine 123 (R123) (0.01 mg/mL) and PI (1 mg/mL) [23]. Briefly, 5 µL of R123 and PI were added to 250 µL of diluted semen (50 × 10^6^ spermatozoa/mL) and incubated for 20 min at 37 °C in the dark. Flow cytometry was performed using a FACSCalibur flow cytometer (Becton Dickinson, San Diego, CA, USA). At least 10,000 events from each sample were recorded at a flow rate of 300–400 spermatozoa/sec. Green fluorescence from R123 was collected through FL1, and red fluorescence from PI was collected through FL3. To determine the correct side scatter and forward scatter of spermatozoa, the samples that were stained with R-123 or PI only and were backgated from the area in which fluorescently stained populations appeared in the green and red channels. The data obtained were analyzed using the CellQuest software (Becton Dickinson, San Diego, CA, USA). The percentage of live spermatozoa with active functional mitochondria was determined by R123 high fluorescence and no PI fluorescence (R123 + /PI-).

#### 2.4.6. Production of Lipid Peroxidation

MDA concentrations in semen samples were measured as an indicator of the lipid peroxidation using the thiobarbituric acid (TBA) test [24]. Each semen sample (500 µL, 25 × 10^6^ spermatozoa/mL) was added to 500 µL of cold 20% (*w/v*) tricholoroacetic, and centrifuged for 5 min at 1200 rpm. The supernatant was mixed with 1 mL of 0.67% (*w/v*) thiobarbituric acid and incubated in a boiling water bath for 10 min. To stabilize the chromogenic MDA-cTBA complex, samples were incubated for 10 min at room temperature. The supernatant was collected and the absorbance was determined at 532 nm by Shima-dzu UV 2100 spectrophotometer [25]. The concentration of MDA was calculated using a standard curve containing known concentrations (0.5–32 µM) of MDA. 

#### 2.4.7. TAC and SOD Assessment

The levels of TAC and SOD were evaluated by commercial colorimetric assay kits (Termo Fisher Scientifc, Waltham, MA, USA) according to the manufacturer’s instructions. TAC was measured by monitoring hydrogen peroxide decomposition. The TAC value was measured using the absorbance at 600 nm, and the concentration was calculated using the calibration curves. The concentration of TAC was expressed as mmol/L. The nitro blue tetrazolium reduction method was carried out to measure SOD activity. Sample concentrations (U/mg) were determined by measuring the mean absorbance values with a pectrophotometer at 630 nm, and converting the SOD activity using a standard calibration curve.

#### 2.4.8. GPx Activity Assessment

GPx activity was evaluated by NADPH oxidation using a coupled reaction system consisting of GSH [26]. In brief, the post-thawed semen of each group (0.1 mL) was mixed with a reaction substance (0.8 mL; 50 mM potassium phosphate buffer, 1 mM sodium azide, 0.2 mM NADPH, and 1 mM GSH). After 5 min of incubation at 25 °C, GPx activity was calculated per the absorbance at about 340 nm by comparing to the calibration curves. The GPx activity was expressed in international units (*IU*) per mg of protein.

### 2.5. Artificial Insemination (AI)

The reproductive performance of the post-thaw semen was evaluated by AI as described previously by Thananurak et al. [23]. According to the results of in vitro sperm assessments, 12 Ross broiler breeder hens (28 weeks old) were separately bred in cages (70 × 70 × 85 cm) and inseminated with frozen–thawed sperm from two experimental groups (n = six hens/each group). The fertility assessments in the SL1.0 and control groups were performed. Sperm was thawed for 5 min in an ice water bath at 5 °C. All hens were inseminated intravaginally according to the following scheme: two days in a row with a single dose of insemination of 0.25 mL of thawed semen 100 × 10^6^ spermatozoa/per hen), and then every tree days. The total number of insemination days was five, and all inseminations were performed between 15:00 h and 16:00 h. Collecting eggs for incubation began a day the after the first insemination and was performed daily for nine days. Eggs (n = 220/per group) were incubated at 37.8 °C in the setter for 18 d and then placed in a hatcher for up to 3 days. Fertility rate (fertilized eggs/incubated eggs × 100%) was evaluated by candling the eggs on day 7 of incubation. After 21 days of incubation, the hatching rate of fertile eggs (hatched eggs/fertilized eggs × 100%) was calculated.

### 2.6. Statistics

A total of three replicates were performed for each in vitro evaluation of sperm parameters. The Statistical Package for Social Sciences (SPSS) V.23 was used for statistical analyses. All data were checked using the Shapiro–Wilk test and were found to fit the normal distribution. The groups were compared using one-way ANOVA, followed by post hoc analysis and the least significant difference test. Differences between two groups were analyzed using Student’s *t*-test. Data in percentages were arcsine-square root transformed before the statistical analysis. The significance level was set to 0.05. Data are presented as mean value ± standard error of the mean (SEM).

## 3. Results

The mean volume, sperm concentration, and pH of fresh sperm from the ejaculate were 1.20 ± 0.26 mL, (742.68 ± 32.12) × 10^6^ spermatozoa/mL, and 6.19 ± 0.18, respectively. The total sperm motility was higher than 80%, the viability was recorded to be more than 90%, and the sperm deformity rate was 7%.

The results of the sperm motility and motion parameters are presented in Table 1. A significantly higher percentage of MOT and higher VAP values were observed in 1% of the nano-SL as compared to the other groups (*p* < 0.05). The percentage of PMOT was significantly higher in the SL0.5 and SL1.0 groups as compared with the other groups (*p* < 0.05). The sperm motility VSL and VCL parameters were significantly higher in the SL0.5 and SL1.0 groups than in the other SL groups (*p* < 0.05). However, differences between the control and the SL1.0 group were nonsignificant (*p* > 0.05). A significantly higher BCF was observed in the SL1.0 group than the other groups (*p* < 0.05). The percentages of LIN and STR were significantly higher in the SL0.5 and SL1.0 groups, as compared to the groups at other SL concentrations and the control groups (*p* < 0.05). For ALH and WOB, no significant differences were found among the groups (*p* > 0.05).

The values of plasma membrane integrity, acrosome integrity, and mitochondrial activity of post-thawed rooster semen are shown in Table 2. The percentages of plasma membrane and acrosome integrities in the SL1.0 group were significantly higher as compared to the other groups (*p* < 0.05). A significant decrease in the percentages of plasma membrane and acrosome integrities were observed in the SL1.5 and SL2.0 groups as compared to the SL0.5 groups (*p* < 0.05). Percentages of sperm with high mitochondrial activity significantly increased in the SL0.5 and SL1.0 groups, as compared to the controls (*p* < 0.05). As regards mitochondrial activity, no significant differences were found between the control and the SL1.5 or SL2.0 groups (*p* > 0.05).

Table 3 presents the effects of SL on the MDA, TAC, SOD, and GPx of post-thawed rooster semen. A significant decrease in the MDA level was observed in the SL0.5 and SL1.0 groups, as compared to the other SL concentration groups and the control groups (*p* < 0.05). A significant increase in TAC activity was observed in the SL1.5 group as compared to the SL2.0 and control groups (*p* < 0.05). However, the value was not different for the SL1.5 group and the SL0.5 and SL1.0 groups (*p* > 0.05). The SOD concentration in the SL1.0 group was significantly higher than that in the other groups (*p* < 0.05). As regards GPx activity, no significant differences were found between the groups (*p* > 0.05).

The results of the fertility and hatching rates after insemination with frozen–thawed semen are listed in Table 4. The percentage of fertility in the SL1.0 group was significantly higher than in the controls (56.36% vs. 42.72%) (*p* < 0.05). It was observed that the hatching rate percentage was significantly higher in the SL1.0 group than in the controls (58.06% vs. 40.43%) (*p* < 0.05).

## 4. Discussion

Semen cryopreservation is a useful tool to preserve genetic resources. These resources are used to prevent extinction by infectious disease, to maintain genetic diversity, and to increase AI success [4]. It is well-known that cryopreservation is responsible for a decrease in the sperm quality, which is largely attributed to physical or chemical damage [27]. For these reasons, a highly effective cryoprotectant and the optimal concentration at which it can be successfully used during semen cryopreservation are of great interest.

Many studies confirm that SL can prevent spermatozoa cryodamage during cryopreservation by mitigating the efflux of cholesterol and phospholipids. Further, it was reported that using 2% nano-SL in the extender improved the sperm cryosurvival of goats, and the results of cryopreservation of sperm are better than any SL suspension [7]. In this study, our objective was to determine the optimal concentration of nano-SL through the quality assessment of frozen–thawed sperm in vitro and in vivo. As far as we are aware, no data are available concerning the influence of nano-SL on the cryopreservation of rooster semen. 

Our findings revealed that among the test concentration range of SL (0.5–2%), the best concentration of SL for cryopreservation of rooster semen was 1.0%. However, previous sperm studies for semen cryopreservation in other species demonstrated that the optimal SL concentration ranged from 0.8 % in canines [9], 1.5% in bulls [8], 2% in rams [7], and 6% in boars [28]. This inconsistency may be related to species-specific differences.

The CASA is an effective, optimized, comprehensive, and detailed technique that is used to assess the quality of sperm in several mammalian species, including humans [29]. Sperm MOT and PMOT are prerequisite factors to determine the fertilizing capacity of spermatozoa in vivo [30]. The values of VAP, VSL, STR, and LIN, as characteristics of sperm velocity over specific paths, have a positive correlation to sperm progression, while VCL, ALH, and BCF have been demonstrated to be effective indicators of sperm vigor [31]. In the present study, 0.5% and 1.0% SL supplementation into the extender improved post-thawed sperm MOT, PMOT, VAP, VSL, VCL, LIN, and STR. These results were consistent with those from previous studies on rams [7], goats [11], and canines [9], which suggests that the lower concentration of SL may lead to higher sperm motion and vigor. 

Our study suggested that high doses of nano-SL had a negative effect on motion characteristics. A similar finding was previously reported in which 1.5% SL added to the extender increased the extender’s viscosity. It was also reported that particular SL debris may reduce sperm motility [32]. Moreover, Nadri et al. [7] reported that the post-thaw sperm motility was decreased when the concentrations of nano-lecithin were higher than 3%, which may due to excessive coating or membrane modifications. Furthermore, our results demonstrated that semen cryopreserved with nano-SL (ranging from 0.5% to 1.0%) had greater BCF values, which is similar to the results of a previous study of canines [9]. This is possibly because the SL in the extender can enhance ATP production and, consequently, the beat frequency [33]. We observed that the addition of nano-SL in the extender had no significant influence on ALH or WOB. This is consistent with previous research that shows that SL in bull semen did not affect ALH or WOB [34].

As the first sperm cell components, membranes are associated with the resistance of sperm to cooling, which is influenced by the chemical composition or temperature changes of the extender [35]. In this study, 1% nano-SL significantly reduced the damage to the cell membrane after thawing as compared with controls; in contrast, significant increases in membrane damage were observed at the nano-SL concentration range of 1.5–2.0%. It was demonstrated that nano-SL infused at a different concentration produces different effects on membrane integrity. Moreover, Mehdipour et al. [32] reported that supplementation of the rooster sperm extender with 1% SL resulted in higher plasma membrane integrity than without SL supplemented in the extenders. It was speculated that SL maintains the stability of cell membranes by preventing the loss of phospholipids and, therefore, protects the membrane via its primary component: phosphatidylcholine [36]. Research in this area also shows that superior semen quality depends on the spermatozoa motility, which results in a directly proportional relationship between motility and membrane integrity values [37].

Semen quality is not only linked to the cell membrane stability but also to acrosome integrity. Acrosomal integrity has a decisive effect on the sperm penetration and fusion ability, which thereby affect the fertility of frozen semen [38]. From our results, 0.5% and 1.0% nano-SL supplementation increased the acrosome integrity as compared with the controls, whereas high concentrations had the opposite effect. It should be mentioned that relatively high concentrations of SL are likely toxic and lead to pro-oxidant effects, exacerbating reactive oxygen species (ROS) production [39]. Similar results were observed in bull semen [40]. On the other hand, a few reports show that there is no significant difference between acrosomal integrity with or without SL exposure in the extenders [6,41]. This shows that the protective role of SL in terms of stabilizing the sperm acrosome varies according to species.

Sperm mitochondria represent the regulatory center of energy metabolism and oxidative stress, and are the hub of signaling pathways that regulate cell survival and apoptosis [42]. In particular, mitochondrial functionality is critical for sperm motility. It was previously reported that exposure of sperms in an ex vivo environment may cause mitochondrial dysfunction [43]. It was reported that the addition of 1% SL to the extender significantly increased mitochondrial activity in post-thawed rooster sperm [32]. In the present study, we showed that nano-SL at doses of 0.5% and 1.0% significantly improved mitochondrial activity. In contrast, SL had a negative effect on mitochondrial activity in frozen–thawed ram semen, as compared with those in egg yolk-containing samples [13]. Therefore, more studies are needed to fully elucidate this mechanism.

Cryopreservation damage of sperm is partly related to ROS and oxidative stress generation. The antioxidant effect of SL has been attributed to phospholipids, phosphatidic acid, lyso-phospholipids, and vitamin E (the main components of lecithins) through multiple mechanisms [36,44]. We next investigated the influence of nano-SL on the enzymatic antioxidant defense system for the cryopreservation of rooster semen. Since MDA is considered an indicator of cell membrane oxidative damage, seminal MDA levels are negatively correlated with seminal antioxidant protection and semen quality [45]. The findings of the present study showed that a significant decrease in the MDA level was observed in the SL0.5 and SL1.0 groups, as compared to the other SL concentrations. Our results are in accordance with those published in previous papers, suggesting that SL may have a beneficial influence on sperm motility through reducing oxidative stress [46]. The results of our study also revealed that the endogenous enzymatic actions of SOD and TAC were enhanced after nano-SL supplementation in the extender, as compared to SL-free extenders. This further illustrates that nano-SL supplementation could have positive effects against oxidative damage due to the freezing-thawed process. Moreover, in the present study, we observed that the GPX activity was not altered during nano-SL treatment, which was similar to a previous study in rams [47]. Nevertheless, it is worth considering that using high levels of nano-SL (ranging from 1.5% to 2.0%) did not have any positive impact in terms of oxidative stress, as compared to the controls. The main reason for this may be that nano-SL, as an antioxidant, has a negative impact due to the excessive scavenging of free radicals, possibly through changing its concentration in the extender [48]. 

The results of our in vitro experiments suggest that a supplementation of 1.0% nano-SL to the extenders is optimum for improving the quality of frozen–thawed rooster semen. Moreover, another main goal of this study was to investigate the impact of nano-SL on sperm function, i.e., on fertility and hatchability rates. Our in vivo results showed that the fertility and hatchability percentages of the eggs were improved by adding 1.0% nano-SL to the extender, as compared with controls. This result further demonstrates that SL had a good effect in terms of improving the post-thaw functionality of rooster semen. According to previous studies, the use of SL nanoparticles may eliminate the toxicity of cryoprotectants, thus helping preserve the spermatozoa functional properties for producing viable offspring [7]. The results suggest that nano-SL supplemented to the extender could be a promising cryoprotectant and have a beneficial effect on the cryopreservation of rooster sperm. 

## 5. Conclusions

In conclusion, a nano-SL dosage of 1.0% in the semen extender had a positive influence on the post-thawing quality in roosters, improving various sperm motion parameters, protecting membrane and acrosome integrities, increasing mitochondrial activity, augmenting antioxidant capacity, and reducing the oxidative stress caused by the cryopreservation process. Moreover, the enrichment of the semen extender with 1.0% nano-SL increased the fertilizing efficiency of rooster sperm after AI. Furthermore, the results from the in vitro and in vivo experiments can serve as a resource for further studies concerning the use of nano-SL as an antioxidant in the extenders for the cryopreservation of rooster semen. We believe that future experiments should assess how nano-SL interacts with spermatozoa and how to preserve or improve this system to achieve the optimum protective efficacy.

## Figures and Tables

**Table 1:** The effect of different concentrations of soybean lecithin (SL) on post-thawed rooster sperm motion parameters (n = 20 roosters and n = 95 ejaculate).

Variables (Unit)	SL Concentration % in Primary Extender ^1^				
	0	0.5	1.0	1.5	2.0
MOT (%)	42.80 ± 1.41 ^b^	45.50 ± 0.52 ^b^	47.30 ± 0. 40 ^a^	31.30 ± 1. 40 ^c^	28.43 ± 0.35 ^c^
PMOT (%)	38.67 ± 1.09 ^b^	42.14 ± 1.15 ^a^	44.94 ± 2.06 ^a^	30.77 ± 1.63 ^c^	26.50 ± 0.93 ^d^
VAP (µm/sec)	37.23 ± 0.68 ^b^	38.51 ± 0.87 ^b^	41.19 ± 0.49 ^a^	29.88 ± 0.99 ^c^	25.31 ± 0.35 ^d^
VSL (µm/sec)	19.51 ± 1.29 ^b^	24.59 ± 1.44 ^a^	22.60 ± 1.61 ^ab^	13.79 ± 1.67 ^c^	12.52 ± 2.98 ^c^
VCL (µm/sec)	55.92 ± 1.31 ^b^	61.86 ± 1.02 ^a^	59.54 ± 0.47 ^ab^	46.09 ± 0.61 ^c^	48.26 ± 3.20 ^c^
ALH (µm)	4.43 ± 0.41	4.10 ± 0.12	4.38 ± 0.27	4.13 ± 0.10	4.22 ± 0.32
LIN (%)	23.70 ± 0.46 ^b^	27.83 ± 2.17 ^a^	29.00 ± 2.15 ^a^	22.14 ± 1.07 ^b^	21.08 ± 2.97 ^b^
STR (%)	56.48 ± 2.89 ^b^	61.27 ± 1.83 ^a^	64.20 ± 1.42 ^a^	45.46 ± 1.75 ^c^	44.38 ± 1.07 ^c^
WOB (%)	52.73 ± 2.02	50.23 ± 1.10	52.84 ± 1.85	50.38 ± 3.42	55.84 ± 3.72
BCF (Hz)	15.40 ± 0.28 ^c^	14.94 ± 0.23 ^c^	18.19 ± 0.69 ^b^	19.04 ± 0.82 ^a^	15.13 ± 0.92 ^c^

^1^ Values with different letters in the same column indicate significant differences *(p* < 0.05).

**Table 2:** Percentages of plasma membrane and acrosome integrities, and mitochondria activity of post-thawed rooster sperm treated with different concentrations of soybean lecithin (SL) (n = 20 roosters and n = 95 ejaculate).

Variables (Unit)	SL Concentration % in Primary Extender ^1^				
	0	0.5	1.0	1.5	2.0
Plasma membrane integrity (%)	39.46 ± 0.87 ^b^	40.62 ± 0.79 ^b^	44.28 ± 0.89 ^a^	36.31 ± 1.15 ^c^	35.13 ± 1.02 ^c^
Acrosome integrity (%)	39.06 ± 0.87 ^c^	45.20 ± 1.41 ^b^	53.16 ± 1.06 ^a^	35.16 ± 1.37 ^d^	36.76 ± 2.20 ^cd^
Mitochondrial activity (%)	41.66 ± 2.94 ^b^	51.09 ± 2.92 ^a^	54.12 ± 1.85 ^a^	37.83 ± 3.12 ^b^	39.85 ± 1.79 ^b^

^1^ Values with different letters in the same column indicate significant differences *(p* < 0.05).

**Table 3:** Malondialdehyde (MDA), total antioxidant capacity (TAC), and superoxide dismutase (SOD) and glutathione peroxidase (GPx) activities of post-thawed rooster sperm treated with different concentrations of soybean lecithin (SL) (n = 20 roosters and n = 95 ejaculate).

Variables (Unit)	SL Concentration % in Primary Extender ^1^				
	0	0.5	1.0	1.5	2.0
MDA (nmol/mL)	2.32 ± 0.11 ^b^	2.14 ± 0.08 ^a^	2.11 ± 0.11 ^a^	2.39 ± 0.09 ^b^	2.30 ± 0.08 ^b^
TAC (mmol/L)	1.19 ± 0.10 ^b^	1.55 ± 0.24 ^a^	1.47 ± 0.22 ^a^	1.59 ± 0.19 ^a^	1.11 ± 0.24 ^b^
SOD (U/mg)	117.48 ± 3.57 ^c^	129.44 ± 1.71 ^b^	141.14 ± 2.13 ^a^	106.80 ± 6.97 ^c^	90.99 ± 2.03 ^d^
GPx (U/mg)	52.75 ± 2.15	51.04 ± 3.36	54.18 ± 5.17	55.48 ± 3.56	51.03 ± 4.10

^1^ Values with different letters in the same column indicate significant differences (*p* < 0.05).

**Table 4:** Evaluation of fertility and hatchability rates of post-thawed rooster sperm treated with soybean lecithin (SL). ^1^

Variables (Unit)	SL Concentration % in Primary Extender ^2^	
	0	1.0
Fertilized eggs (N)	94	124
Fertilized eggs (%)	42.72 ± 4.35 ^a^	56.36 ± 3.93 ^b^
Hatched eggs (N)	38	72
Hatched eggs ratio (hatched/fertilized, %)	40.43 ± 3.78 ^a^	58.06 ± 4.67 ^b^

^1^ Number of eggs per group = 220. ^2^ Values with different letters in the same column indicate significant differences *(p* < 0.05).
